# Effect of Extrusion Conditions on the Characteristics of Texturized Vegetable Protein from a Faba Bean Protein Mix and Its Application in Vegan and Hybrid Burgers

**DOI:** 10.3390/foods14040547

**Published:** 2025-02-07

**Authors:** Maria Guerrero, Andrea K. Stone, Ravinder Singh, Yuk Chu Lui, Filiz Koksel, Michael T. Nickerson

**Affiliations:** 1 Department of Food and Bioproduct Sciences, University of Saskatchewan, Saskatoon, SK S7N 5A8, Canada; cbo542@mail.usask.ca (M.G.); andrea.stone@usask.ca (A.K.S.); sara.lui@usask.ca (Y.C.L.); 2 Department of Food and Human Nutritional Sciences, Richardson Centre for Food Technology and Research, University of Manitoba, Winnipeg, MB R3T 2N2, Canada; singhr9@myumanitoba.ca (R.S.); filiz.koksel@umanitoba.ca (F.K.)

**Keywords:** protein isolate, protein concentrate, extrusion, texture profile analysis, pulse protein, meat alternative

## Abstract

The aim of this study was to produce texturized vegetable proteins (TVPs) from faba bean protein via low-moisture extrusion. The effect of extrusion variables including temperature (110, 125, and 140 °C at the die), feed moisture content (30, 35, and 40%), and screw speed (200, 300, and 400 rpm) on the TVP properties were investigated. An increase in feed moisture content or extruder temperature reduced the specific mechanical energy and torque by 40–45% during extrusion. An increase in feed moisture created TVPs with lower bulk densities and rehydration ratios while an increase in extruder temperature or screw speed increased the bulk density of the TVPs. An increase in screw speed also caused a decrease in the water holding capacity of the milled TVP flours. The TVP flours had a 33–70% higher oil holding capacity than the raw material. The texture profile showed that an increase in feed moisture influenced TVP hardness, gumminess, and chewiness with higher values compared to the treatments with lower moisture contents. Springiness, cohesiveness, and resilience were more affected by a change in screw speed with higher values at 200 rpm. The best parameters were selected (125 °C, 40% MC, 300 rpm) to produce TVP to use as a partial (hybrid burger) and complete (vegan burger) replacement of beef in a burger patty. The replacement of 25% beef with TVPs in a hybrid burger increased the cooking yield and moisture retention and decreased the thickness and diameter change compared to the beef burger without TVPs. In a vegan formulation, the faba bean TVP burger had lower cooking yield and moisture retention than commercial products.

## 1. Introduction

Meat is considered a high-quality protein because of its complete amino acid profile, and it has an appearance, texture, and mouthfeel that is highly accepted by consumers [1]. However, meat sources have health and sustainability concerns such as a high content of saturated fats and the limitation of resources including water and land [2]. Many consumers are moving towards a more sustainable and healthier lifestyle with high acceptance of vegan, vegetarian, and hybrid (partial meat replacement) products creating a new space for innovation in the plant-based market [3]. Meat replacements are part of these products that use plant proteins to mimic the functionality, taste, texture, and look of real meat while also often having specific health and environmental benefits [4]. The growing interest in plant-based alternatives is creating an increased need for research studies to develop new products to increase food diversification [5].

Pulse proteins (i.e., lentil, pea, faba bean, etc.) are being adopted as high-quality raw materials to produce meat alternatives due to their broad range of functionality, low-fat content, and good protein content. Pulse consumption is also related to health benefits like lowering the risk of type 2 diabetes, cardiovascular diseases, and cancer [6]. Faba bean (*Vicia faba*) specifically is experiencing widespread market growth due to its superior protein content (25–40%) compared to other pulses (peas, chickpeas, and lentils) [7,8]. Furthermore, Canada, among other countries, is focusing on faba bean breeding efforts to increase yield and productivity and decrease anti-nutritional factors like tannins and vicine/convicine content [9].

Low-moisture (<40% moisture content) extrusion technology can be used to produce plant-based extrudates that have fibrous structures and that give good functional properties including high water holding capacity and rehydration ratios [10]. Upon rehydration, these low-moisture extrudates form sponge-like structures and have textural attributes that mimic animal tissue texture [11]. Faba bean protein has previously been used in high-moisture extrusion (>40% moisture content) to create meat analogs [12,13] but has had limited success as the sole ingredient in low-moisture extrusion [14]. Extrusion improves the native protein structure through denaturation that exposes certain amino acids. With this technology, it is possible to produce higher-quality products that maintain or increase the nutritional benefits of pulses while also removing the anti-nutritional factors [15].

The combination of different variables during extrusion, such as moisture content (of the feed material), barrel temperature profile, and screw speed, produce texturized vegetable proteins (TVPs) that are rehydrated and used as a partial or complete substitution for meat in products such as burger patties, meatballs, sausages, nuggets, and others [11,16]. Of the different categories of plant-based meat analogs, burgers had the highest level of consumption in 2023, and the plant-based burger market in North America is expected to swiftly advance in size and value over the coming years [17]. When used as a partial meat replacement in burgers, TVPs act to increase cooking yield, water holding capacity, and protein content while also decreasing the costs of production. The addition of TVPs in burgers can also have a positive impact on textural properties (hardness and cohesiveness) and improve appearance attributes including color and shape [18].

TVPs are traditionally made from soy and are not novel products; however, faba bean protein ingredients have only recently begun to be investigated for TVP production, and, thus, the faba bean TVP is a novel ingredient to produce and evaluate [19]. The aim of this study was to evaluate the physical and functional properties of TVPs from faba bean protein (isolate and concentrate) through low-moisture extrusion using different barrel temperature profiles (110, 125, and 140 °C at the die), feed moisture contents (30, 35, and 40% or g water/100 g feed), and screw speeds (200, 300, and 400 rpm). The best condition was then chosen to produce faba bean-based TVPs to partially (hybrid burgers) or completely (vegan burgers) replace ground beef in burger patties. TVP burgers were compared to beef burgers or commercial plant-based burgers. It is hypothesized that higher specific mechanical energy, induced by higher screw speeds, elevated temperatures, and lower moisture, will result in more fibrous TVPs.

## 2. Materials and Methods

### 2.1. Materials and Protein Content

Faba bean protein isolate (92.4% protein, db) and concentrate (60.5% protein, db) were provided by AGT (Alliance Grain Traders) Food and Ingredients (Regina, SK, Canada). Protein content was determined by measuring the nitrogen contents (conversion factor of 6.25) of the samples using the Dumas combustion method with a nitrogen/protein analyzer (CN628, LECO Corporation, St. Joseph, MI, USA) (AACC International Method 46-30.01) [20]. Upon arrival, the feed material was stored at 4 °C. The faba bean ingredient mix used as the feed material for extrusion in this study was 70% faba protein isolate and 30% faba bean protein concentrate (82.8% protein, db). Commercial pea TVPs were sourced for comparison purposes, including CTVP-1 (80.6% protein, db) and CTVP-2 (75.2% protein, db).

### 2.2. Extrusion

The relevant amount of boiling water was added to the feed material to pre-condition it to 26.5 ± 0.5% (wet basis) a day prior to the extrusion run. The pre-conditioning was performed by mixing the feed material in an A200 Hobart mixer (Hobart, Offenburg, Germany) at a low speed for 5 min while the boiling water was added. Immediately after mixing, the material was packed in zipped polyethylene plastic bags and stored in a climate chamber (HPP 260 IPP plus, Memmert, Schwabach, Germany) at a constant temperature (50 °C) until extrusion. The TVP was produced using the pre-conditioned material using a twin-screw (co-rotating) lab-scale extruder (MPF19, APV Baker Ltd., Peterborough, UK) using a circular die of 2.3 mm diameter. The screw length-to-diameter ratio was 25:1. The feed rate was kept constant at 2.75 kg/h (dry basis). The screw configuration is given in Singh et al. [16]. Extrudates were collected at a combination of different barrel temperature profiles (110, 125, and 140 °C at the die), feed moisture contents (MCs; 30, 35, and 40% or g water/100 g wet feed), and screws speeds (200, 300, or 400 rpm), as shown in Table 1. For all treatments, TVPs were collected in triplicate (i.e., three separate extrusion runs). Values for torque and die pressure were collected in quadruplicates for each extrusion run, the average of which was taken as the value for that run. Specific mechanical energy (SME) was calculated as follows:

(1) $$SME\left(\frac{Wh}{kg}\right)=\frac{\frac{actual\;screw\;speed}{\max screw\;speed}\times \frac{torque}{100}\times motor\;power}{mass\;flow\;rate}$$

where the max screw speed was 500 rpm, and the motor power was 2200 W.

After extrusion, the TVPs were dried for up to 24 h at ambient temperature followed by drying in an air oven (Isotemp 180 L Oven Gravity, Thermo Scientific, Langenselbold, Germany) for up to 72 h at 40 °C to reduce their moisture content below 10% for long-term storage (actual values, 6–7%). A subsection of the dried TVPs was milled into a flour/powder (Retsch, ZM 200, Haan, Germany). The intact TVPs and TVP flours were then placed in zipped plastic bags and stored at 4 °C for further analysis.

### 2.3. Composition and Physical Properties

Protein content was determined as previously described. Moisture content was determined according to AOAC method 925.10 [21]. Water activity (a_w_) of the samples was conducted with an AquaLab 4TE water activity meter (Decagon Devices, Inc., Pullman, WA, USA) at 22 °C. Color was measured using a Hunter Colorimeter (ColorFlex EZ 45/0, Hunter Associates Laboratory, Inc., Reston, VA, USA). The L * (lightness), a * (redness), and b * (yellowness) values are reported. All measurements were performed in triplicate. The results are reported as the mean ± one standard deviation. Scanning electron microscopy (SEM) was used to examine the microstructure of the materials according to the method of Koksel and Masatcioglu [22]. In brief, the sample was placed on aluminum stubs with silver paste and coated with a Au-Pd alloy at a thickness of 20 nm using a cold sputter coater (Denton Vacuum, Desk II, Cranford, NJ, USA). The images were obtained using the SEM (Quanta FEG 650, FEI, Hillsboro, OR, USA) with an acceleration voltage of 3 kV at 500× magnification for the TVPs and a range of 250× to 900× for the faba bean ingredient mix used as the feed material for extrusion (i.e., 70% faba protein isolate + 30% faba bean protein concentrate).

### 2.4. Functional Properties

Water and oil holding capacities were measured by weighing 0.5 g (protein weight basis—adjusted for protein content of samples) (W_1_) of milled TVP flour or the raw faba bean ingredient mix into a 50 mL centrifuge tube followed by 5 g of distilled deionized water or canola oil, respectively. Tubes were vortexed for 10 s every 5 min for 30 min at the maximum speed of 10 (Analog Vortex Mixer, VWR International, Mississauga, ON, Canada) and then centrifuged at 1000× *g* for 15 min (Sorvall ST8 Centrifuge, Thermo Fisher Scientific Inc., Waltman, MA, USA). The supernatant was drained, and the mass of the resulting pellet was weighed (W_2_). The amount of water or oil absorbed was determined based on differences in mass prior to and after analysis (report as g water or oil/g sample). WHC/OHC were calculated using the following equation:

(2) $$WHC/OHC(g/g)=\frac{W_{2}-W_{1}}{W_{1}}$$

The bulk density of the TVPs was measured by adding the TVPs into a graduated cylinder with a defined volume. Bulk density was calculated as the weight (g) of the TVPs divided by the sample bulk volume (mL) of the graduated cylinder following the method described by Lisiecka et al. [23].

The rehydration ratio (RR) was calculated according to Brishti et al. [24] and Yu et al. [25] with some modifications. A sample of TVP (W_1_: ~40 g for faba bean TVPs and 15 g for commercial TVPs) was combined with 1000 mL of distilled water at 80 °C for 30 min. Water was then drained through a 60-mesh screen for 10 min, and the weight of the rehydrated samples was recorded (W_2_). The rehydration ratio was calculated using the following equation:

(3) $$RR(\%)=\frac{W_{2}-W_{1}}{W_{1}}\times 100$$

All functional measurements were performed in triplicate. The results are reported as the mean ± one standard deviation.

### 2.5. Texture

The texture profile of the TVPs was measured using a texture analyzer (Texture Technologies Corp., South Hamilton, MA, USA) with Probe TA-4 (contact area 1140 mm^2^) according to Brishti et al. [24] with some modifications as described in Singh et al. [16]. In brief, rehydrated TVPs (prepared as described for the RR) were placed in a Petri dish (height of 15.6 mm). TVPs were then pressed to 50% of the original height at a speed of 1 mm/s to give hardness (peak force), and springiness, cohesiveness, gumminess, chewiness, and resilience values were calculated by the software. Measurements were performed in triplicate and the mean value of six measurements was used for each replicate. The results are reported as the mean ± one standard deviation.

### 2.6. Hybrid Burger Preparation

One faba bean-based TVP was chosen to test in burger formulations, T4, with extrusion conditions of 125 °C, 30% MC, and 300 rpm. A hybrid burger formulation was developed to allow the partial substitution of ground beef with TVPs (Table S1). Control beef burgers contained 91% lean ground beef (16% fat), 7.7% water, 1% salt, and 0.3% black pepper. Hybrid burgers contained 68.15% lean ground beef (16% fat), 10% TVP (by weight before rehydration), 15.3% water, 1% salt, 0.3% black pepper, 4.4% canola oil, and 0.85% food color and flavor.

The TVP (faba bean-based or commercial) was rehydrated by placing it in the bowl of a kitchen stand mixer (with a paddle attachment) with water (1:1.5, TVP/water, *w*/*w*) for 10–15 min (until all water was absorbed) with occasional mixing. The remaining water required for the formulation and the food color and flavor were then added and mixed for 30 s. The remaining ingredients (salt, pepper, oil, and ground beef) were added during mixing. Patties weighing 113 g were shaped with a manual patty press (height: 1.2 cm; diameter: 10.5 cm) and then covered with plastic film and frozen at −40 °C for at least 24 h. Frozen burgers were cooked on a preheated (163 °C) electric pan to an internal temperature of 73.8 °C. Burgers were prepared in triplicate.

### 2.7. Vegan Burger Preparation

The same faba bean-based TVP (T4) used in the hybrid burgers was used in the vegan burgers. Vegan burgers contained 20% TVP (by weight before rehydration), 60% water, 1% salt, 0.3% black pepper, 13% canola oil, 3.2% food color and flavor, and 2.5% methylcellulose (Table S1). A commercial plant-based patty (Beyond Burger^®^, Beyond Meat, El Segundo, CA, USA) was purchased to compare it with the vegan TVP burgers. The beyond burger was selected based on its widespread use in the North American market.

The TVP was rehydrated as previously stated for the hybrid formulation. The remaining water, methylcellulose, and food color and flavor were then mixed with the rehydrated TVP followed by the addition of the canola oil, salt, and pepper during mixing. Burger patties were shaped, frozen, and cooked in triplicate as previously described.

### 2.8. Burger Properties

The texture profile of the cooked burgers was measured at room temperature (21–22 °C) using the same texture analyzer and probe as previously mentioned. Each burger was pressed to 25% of its original thickness at a speed of 1 mm/s to obtain hardness, springiness, cohesiveness, gumminess, chewiness, and resilience values. Measurements were performed in triplicate, and the mean value of six measurements was taken for each replicate.

The cooked burgers were allowed to reach room temperature (21–22 °C) before measurement of the cooking properties. Cooking yield (%) was measured by dividing the cooked burger weight by the raw burger weight. The difference between the diameter of the raw and cooked patty divided by the raw patty diameter is given as diameter change (%). Thickness change (%) was similarly calculated except using the raw and cooked patty thickness instead of diameter. To obtain a single replicate value a total of four thickness measurements per patty were made. Moisture retention (%) was calculated by measuring the moisture content of the cooked patties after drying at 105 °C for 18 h.

(4) $$Moisture\;retention\left(\%\right)=\frac{Cooking\;yield\left(\%\right)\times Moisture\;of\;cooked\;patty\;(\%)}{100}$$

Measurements were made in triplicate for each sample, and the results are reported as the mean ± one standard deviation.

### 2.9. Statistical Analysis

The data were analyzed using Minitab statistical software (22.2, MINITAB Inc., State College, PA, USA) with one-way analysis of variance (ANOVA) and Tukey tests. A probability of *p* ≤ 0.05 was used to determine a significant result.

## 3. Results and Discussion

### 3.1. Extrusion Response Parameters

Specific mechanical energy (SME) was calculated based on the torque, screw speed, motor power, and mass flow rate for each treatment to produce faba bean-based TVPs where values ranged from 72.0 to 118.4 Wh/kg (Table 2). Torque values ranged from 23.3% to 56.9%, where there was a tendency to have lower values with higher temperatures as shown in Table 2. For T6 at 140 °C (35% MC, 300 rpm), the torque value decreased by 10% compared to T7 at 110 °C with the same feed MC and screw speed (i.e., 35% MC, 300 rpm). This is attributed to the change in viscoelastic properties of the material, i.e., a drop in viscosity, associated with higher mobility at elevated temperatures [26]. Temperature also impacted the SME where T6 at 140 °C (35% MC, 300 rpm) was 31.9 Wh/kg lower compared to T7 at 110 °C (35% MC, 300 rpm). Similar results were obtained by Saldanha do Carmo et al. [19] where SME values decreased from 557.7 to 520.4 Wh/kg when the temperature was increased from 140 °C to 160 °C with 30.8% MC and 900 rpm for blended faba bean protein concentrate–oat fiber extrudates. For the MC, there was a decrease in SME from 109.16 to 72.0 Wh/kg when the MC increased from 30% (T4: 125 °C, 300 rpm) to 40% (T5: 125 °C, 300 rpm) which is attributed to the decrease in viscosity of the material. Singh et al. [16] also reported SME to decrease as the MC or temperature increased during the production of lentil protein isolate TVP. Finally, screw speed had mixed effects on SME. The increase from 300 rpm (T2) to 400 rpm (T3) at the same MC and temperature (35% MC, 125 °C) created a ~17% increase in the SME. This was hypothesized to be due to a higher amount of friction generated at the higher screw speed. However, the highest SME was recorded using a lower screw speed of 200 rpm (T1) (35% MC, 125 °C) which is attributed to the low mobility of the material based on a longer residence time. Previous studies on the low-moisture extrusion of faba, lima, pinto, and red kidney bean flours showed that a screw speed lower than 250 rpm increased the SME and torque for all the bean types studied [27].

### 3.2. Composition and Physical Properties

The combination of pressure, moisture, temperature, and shear in the extruder creates a mass that traps water where air bubbles are generated, resulting in porous elongated structures due to the change in pressure at the die [28]. To confirm the texturization of the faba bean material, the raw faba bean ingredient mix and the TVPs were imaged by scanning electron microscopy. The raw faba bean ingredient mix had a globular structure (Figure S1A–C) whereas for all extrusion treatments, the network changed to elongated structures that were fibrous in nature (Figure S1D–L). The TVPs produced using different extrusion conditions could not be visually differentiated from each other via scanning electron microscopy images.

The protein content and water activity of the TVPs are given in Table 3. As expected, there was no protein loss during extrusion as the raw faba bean ingredient mix contained 82.8% protein and the TVPs contained 82.7–84.1% protein. The commercial pea TVPs used for comparison purposes in this study were lower in protein at 80.6% (CTVP-1) and 75.2% (CTVP-2). Water activity can be related to the microbial stability of the product since it determines the growth of microorganisms related to spoilage. All the TVPs had water activity values lower than 0.25 (Table 3), creating a stable product where the possibility of growing molds, yeast, or bacteria is low. Water plays a key role in the quality and stability of foods since it can act as a reaction medium for other molecules that could create unwanted flavors or odors that decrease the product’s shelf life. Comparatively, the commercial pea TVPs had water activities that were significantly lower than lab-produced faba bean TVPs with values of 0.15.

### 3.3. Color

The Maillard reaction is a non-enzymatic reaction that leads to browning pigments. The reaction involves a free amino acid (lysine) from proteins and a carbonyl group from the reducing sugars present in carbohydrates to create the changes in color that occur in the presence of higher temperatures. The use of high barrel temperatures and low feed moisture favors the Maillard reaction because of the protein denaturation that can lead to free lysine and the availability of reducing sugars due to the hydrolysis of starch. As shown in Table 3 there was a change in the color for faba bean-based TVPs where brightness values (L *, 68.2–71.6) for all treatments were significantly lower compared to the raw faba bean ingredient mix (L *, 86.5). For redness (a *), the values more than doubled after extrusion for all treatments, and for yellowness (b *), values increased by 1.8–2-fold, both of which can be attributed to the pigments created by the non-enzymatic reaction.

The changes in moisture from 30% (T4) to 40% (T5) (both 125 °C, 300 rpm) slightly increased the TVP brightness, coupled with decreasing the a * and b * values. This is due to the rate of Maillard reactions directly as it relates to the intensity of the extrusion process (i.e., more intense at lower MCs). Moreover, the lubricating effects of water at higher MCs reduce shearing forces within the barrel, potentially causing lower starch degradation [29]. With higher starch fragmentation at lower MCs, more carbonyl groups from the reducing sugars may become available for the Maillard reaction, causing the darker color of the TVPs. Singh et al. [16] also reported the same trend in lentil TVP color based on MCs. An increase in temperature also led to a darker product; there was a decrease in brightness and an increase in redness and yellowness when the temperature was increased from 110 °C (T7) to 140 °C (T6) (both 35% MC, 300 rpm).

There was no change in brightness based on screw speed; however, there was an increase in a * and b * values as screw speed increased from 200 rpm (T1) to 400 rpm (T3) at 35% MC and 125 °C. Previous studies showed that the browning index was affected by higher temperatures (140 °C) and lower moisture contents (46%) which promoted the browning reactions for faba bean concentrate meat analogs produced by high-moisture extrusion [12]. Finally, the commercial pea TVPs were comparable to the lab-scale faba bean-based TVPs but were overall brighter (higher L * value) and redder (higher a * value).

### 3.4. Water Holding Capacity

Water holding capacity (WHC) is associated with the protein matrix, as well as the more minor components (starch and fiber), and relates to how much water the ingredient can bind against a defined force (centrifugation) [28]. For this study, the WHC was determined on the TVP extrudates after they were milled into a flour/powder. Faba bean-based TVP flours had WHC values ranging from 2.95 to 3.78 g water/g of protein (Table 4). The raw faba bean ingredient mix had a WHC of 3.17 g/g, and all faba bean TVP flours were similar to this except for T1 (125 °C, 35% MC, 200 rpm) which produced TVPs with a significantly higher WHC at 3.78 g/g. There was a 12.7% decrease in WHC when screw speed increased from 200 rpm (T1: 125 °C, 35% MC) to 400 rpm (T3: 125 °C, 35% MC). This is hypothesized to be caused by a) shear-induced protein aggregation where hydrophilic sites became more positioned in the interior and b) increased protein–starch interactions, resulting in less starch binding to water [30]. Previous studies show extrudates produced from pea and oat protein with low-moisture extrusion (25–35% MC, 135–160 °C, and 200–1200 rpm) ranged from 1.6 to 2.5 g water/g of sample [31]. While there was no significant difference in WHC between the TVPs using the lowest (T4) or highest (T5) feed MCs (30 and 40%, respectively; both 125 °C, 300 rpm), at the 35% MC (T2), the TVP flour had significantly higher WHC (an increase of ~0.5 g/g). Similarly, although the TVPs produced using the lowest (T7, 110 °C) and highest (T6, 140 °C) temperatures (both 35% MC, 300 rpm) had similar WHC, the TVP produced using the midrange temperature (T2, 125 °C) had 12% higher WHC than the lower temperature TVP (T7). In contrast, Singh et al. [16] reported the trends of increased screw speed and decreased MC to increase the WHC of lentil protein TVP flour. Finally, the commercial pea TVP flours generally had higher WHC values (3.9 and 4.1 g water/g of protein) than the faba bean TVP flours.

### 3.5. Oil Holding Capacity

The oil holding capacity (OHC) relates to the interactions between hydrophobic amino acids on the protein surface and oil particles. In general, the OHC of any extrudate tends to increase compared to the raw material [10], due to the partial unfolding of the protein structure by denaturation exposing more hydrophobic amino acids [32]. The OHC of the raw faba bean ingredient mix, at 0.99 g of oil/g protein, was significantly lower than the OHC of the TVP flours regardless of extrusion treatment conditions. This was in line with the findings reported for pea protein-based TVPs [10]. The TVP flours had OHCs ranging from 1.32 to 1.69 g/g. The T4 (30% MC, 125 °C, 300 rpm) TVP flour had a significantly lower OHC content than T2 (35%, 125 °C, 300 rpm) which used a higher feed moisture content. Similarly, Singh and Koksel [33] reported an enhancement in the OHC of extruded soybean meal when the MC was increased from 15 to 27% and attributed these findings to the increased exposure of non-polar amino acids at higher MC levels. However, under the same temperature and screw speed conditions, the highest MC of 40% (T5) produced TVP flour with the lowest OHC. This is attributed to the changes in melt viscosity; since more moisture is available, the material moves through the barrel in a shorter period, and this reduces the exposure to heat and, as a consequence, the denaturation of the protein [19]. Previous studies showed that the use of faba bean concentrates in high-moisture extrusion decreased the OHC by around 20% compared to the native material due to the aggregation of proteins that decreased the surface hydrophobicity [12]. The OHC of the TVP flour was stable as the barrel temperature increased from 110 °C (T7: 35% MC, 300 rpm) to 125 °C (T2: 35% MC, 300 rpm); however, further increasing the temperature to 140 °C (T6: 35% MC, 300 rpm) resulted in a decrease in TVP flour OHC. This is hypothesized to be due to excessive protein aggregation at the higher temperature decreasing the access to hydrophobic binding sites. Similar results were observed by Wang et al. [34] for chickpea flour where a change from 120 °C to 150 °C (24% MC, 317 rpm) created a small decrease in OHC from 1.3 to 1.2 g oil/g flour. No changes were observed for an increase in temperature from 110 °C to 125 °C (35% MC, 300 rpm) due to the thermal stability of the storage proteins (i.e., vicilin and legumin) present in the faba bean mix with denaturation temperatures higher than 105 °C [35]. The OHC of the TVP flour was not affected by the change in extrusion screw speed, which can be attributed to the large number of entanglements present that reduces the degradation caused by shear [30]. Singh et al. [16] reported the OHC of lentil TVP flour to be higher than the raw material (lentil protein isolate); however, there were no trends based on extrusion conditions (MC, screw speed, or temperature). CTVP-1 flour had a similar OHC to many of the faba bean TVP flours; however, CTVP-2 had a much higher OHC of 2.8 g/g.

### 3.6. Rehydration Ratio

The rehydration ratio (RR) can be defined as the amount of water absorbed by the intact TVP structure after submersion in water (for a given time and temperature), which can affect texture, juiciness, and other sensory properties [18,25,36]. This functional property is affected by the interactions between protein and water, the TVP bulk density, and the porous structure created by the different extrusion variables [18]. For faba bean-based TVPs, the RR had high variability, ranging from a low of 155% to a high of 309% (Table 4). An increase in MC from 30% (T4) to 35% (T2) and then to 40% (T5) (all 125 °C, 300 rpm) created a decrease in the TVP RR from 309% to 280% and finally to 155%. This is attributed to the porosity of each treatment and the interaction between protein and water during denaturation. Similar results were observed by Samard et al. [18] where the RR values ranged from 217 to 367% with the lowest RR obtained at 40% MC and 130 °C for TVPs consisting of a blend of soy protein–wheat gluten–corn starch. No effect was observed with an increase in temperature from 110 °C (T7, RR 235%) to 140 °C (T6, RR 238%) at 35% MC and 300 rpm or a change in screw speed from 200 rpm (T1, RR 297%) to 400 rpm (T3, RR 290%) at 125 °C and 35% MC for the faba bean-based TVPs. In contrast, Singh et al. [16] reported minor improvements in the RR of lentil TVPs as screw speed increased (300 to 450 rpm) or as MC (30 to 40%) increased. Overall, the RRs of the faba bean-based TVPs in the current study, with the exception of T5, were higher than the lentil TVPs in the aforementioned study (RR 182–215%). Finally, the RR values for the commercial pea TVPs were 100–250% higher than the lab-scale faba bean-based TVPs. One reason for this is hypothesized to be the high porosity of the commercial materials as shown by their low bulk density (BD) values (discussed below). Yu et al. [25] reported a negative correlation between RR and BD values for corn flour–soy protein isolate extrudates; however, the faba bean TVPs in the current study did not follow the same trend.

### 3.7. Bulk Density

Bulk density (BD) is a physical property that considers the expansion in all directions caused by extrusion. There are many factors that can affect BD including raw material composition, particle size, moisture content, extrusion conditions, and others [30]. For faba bean-based TVPs, BD values ranged from 0.30 to 0.42 g/mL (Table 4). At higher MCs, lower BD values were produced; an increase from 30% (T4: 125 °C, 300 rpm) to 40% MC (T5: 125 °C, 300 rpm) decreased TVP BD from 0.41 to 0.30 g/mL. The same trend was reported by Singh et al. [16] for lentil protein isolate TVP who attributed it to the higher moisture of the feed material decreasing the melt viscosity inside the extruder barrel. The expansion and porosity of the material are commonly negatively correlated to its bulk density [37]. An increase in temperature from 110 °C (T7: 35% MC, 300 rpm) to 140 °C (T6: 35% MC, 300 rpm) created an increase in BD from 0.34 to 0.42 g/mL. This can be attributed to a higher degree of polymerization and enhanced texturization [30]. There was also a slight but significant increase in BD from 0.36 to 0.39 g/mL when the screw speed changed from 200 rpm (T1) to 400 rpm (T2) (25 °C, 35% MC). In contrast, an inverse relationship between TVP density and extruder screw speed has been reported for TVP from hempseed protein concentrate [38], lentil protein isolate [16], and pea protein concentrate [39]. The authors attributed this to high shear increasing the porosity and expansion of the material upon leaving the die and therefore reducing the TVP density. The commercial pea TVPs had significantly lower BD values than the faba bean-based TVPs, especially CTVP-2 (BD, 0.11 g/mL).

### 3.8. Texture Profile

Protein unfolding is enhanced in the barrel due to high temperatures and shear followed by crosslinking that takes place at the die since proteins are aligned by shear and changes in pressure which is attributed to the desired texture of TVPs [30]. Hardness is part of the texture profile that is related to the peak force needed to compress the TVP. Cohesiveness measures the durability of the internal bonds while springiness measures sample recovery after deformation. Chewiness measures the force that is needed to chew solid food until it is ready to be swallowed, while gumminess measures the viscoelastic properties. Resilience measures how fast and strong the sample recovers after compression. As shown in Table 5, hardness, chewiness, and gumminess values increased when MCs increased from 30% (T4: 125 °C, 300 rpm) to 40% (T5: 125 °C, 300 rpm) whereas springiness and cohesiveness values decreased. The water content changes the viscosity and elasticity inside the barrel during extrusion and therefore the texture properties of the TVP extrudate. Furthermore, as TVP-T4 had twice the RR as T5, it is expected that this extra moisture would allow the extrudate to become softer (i.e., lower hardness). In contrast to the current study, Kaleda et al. [31] attributed a higher hardness to less expansion of the material; however, their starting formulation was a blend of oat protein concentrate and pea protein isolate. A change in screw speed from 200 rpm (T1: 125 °C, 35% MC) to 400 rpm (T3: 125 °C, 35% MC) was associated with lower cohesiveness and chewiness values; however, there was no difference between the hardness, springiness, or gumminess values of T1 and T3. In contrast, Singh et al. [16] reported increased screw speed (from 300 to 450 rpm) to increase lentil TVP springiness, cohesiveness, and resilience; these texture attributes were also increased at lower MCs (30% vs. 40%). Similarly, for texturized hempseed protein, hardness, springiness, chewiness, and cohesiveness all increased in parallel with a decrease in MC or an increase in screw speed [38]. There was no effect on the TVP texture with an increase in extruder barrel temperature from 110 °C (T7: 35% MC, 300 rpm) to 140 °C (T6: 35% MC, 300 rpm). Similarly, Singh et al. [16] did not find any meaningful trend in lentil TVP texture based on temperatures ranging from 120 to 140 °C and further reported that the hardness and chewiness of the TVP could not be associated with the variable extrusion parameters used. The cohesiveness values ranged from 53.9% to 63.0% for faba bean-based TVPs, which is related to the protein–protein interactions where an increase in the value represents a higher number of protein–protein interactions. Similar values were reported by Kaleda et al. [31] who showed a positive association between protein concentration and both cohesiveness and springiness for blends of oat protein concentrate and pea protein isolate TVPs where values decreased when there was less pea protein in the blend. Saldanha do Carmo et al. [19] reported cohesiveness and SME to be highly correlated for TVPs consisting of faba bean protein concentrate and oat (beta-glucan-rich fraction) which the authors related to the enhanced protein interactions and crosslinking. In the current study, the treatments with the highest and lowest SME (T1 and T5, respectively) correspond to the TVPs with the highest and lowest cohesiveness; however, not all of the remaining treatments followed the same trend. For resilience, there was a slight decrease from 33.9% to 30.8% when screw speed increased from 200 rpm (T1) to 400 rpm (T3), at 125 °C and 35% MC. Compared to the commercial pea TVP, faba bean TVPs had higher hardness, gumminess, and chewiness values but lower springiness, cohesiveness, and resilience values.

### 3.9. Application of TVP in Burgers

One of the main characteristics that affect the final sensory aspects of a meat product is the ability to retain liquid after cooking which is directly related to the juiciness and tenderness of the patty. Based on the data collected for faba bean-based TVPs, the best treatment selected for application was T4 (125 °C, 30% MC, 300 rpm) as it had the highest RR out of the seven treatments and a balanced texture profile in relation to the commercial TVPs.

### 3.10. Cooking and Texture Properties for Hybrid Burgers

**Figure 1:**
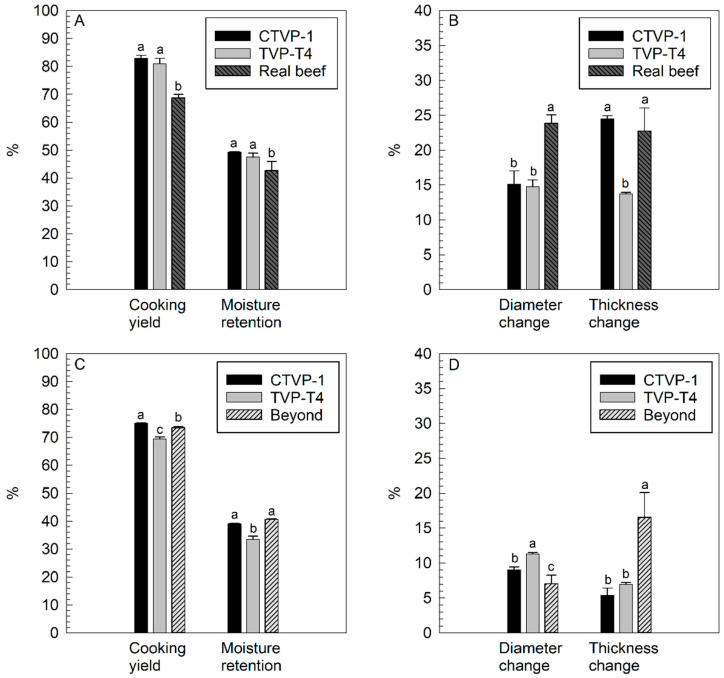
Cooking properties of hybrid (**A**,**B**) and vegan (**C**,**D**) burgers containing commercial TVP (CTVP-1) or faba bean TVP (TVP-T4: 125 °C, 30% moisture content, 300 rpm). Beyond = commercial beyond burger. Samples with the same superscript letter within each property are not significantly different (*p* > 0.05).

The cooking properties of the hybrid TVP–beef burgers and the control beef burger are presented in Figure 1A. Cooking yield measures the capability of the patty to retain water and other juices before and after cooking [40]. The addition of 10% TVP, corresponding to a reduction in the beef content by 25% in the formulation, significantly increased the cooking yield from 68.7% for the real beef burger to >80% for burgers containing commercial pea or lab-scale-based faba bean TVPs. This relates to the moisture retention of the burger where a similar trend was found in that the TVP-containing burgers had higher moisture retention values (47–49%) compared to the real beef burger (42.7%) (Figure 1A). Loss of moisture during cooking can be associated with the contraction of collagen due to heat that enhances liquid expel, meaning that substituting a portion of the meat with TVP that better retains liquid will, as a consequence, reduce the loss of moisture [14]. Previous studies showed similar results where the addition of 30% hydrated texturized whey protein decreased burger cooking loss by 6 percentage points [40]. Gujral et al. [41] reported cooking loss to decrease by 28 percentage points when soy TVP was added at the 20% level to goat meat burgers due to the high moisture retention of TVP. Similarly, Chan et al. [10] reported a 2.7–13.3 percentage point decrease when pea TVP was added at the levels of 20–40% to beef burgers. Carvalho et al. [42] reported that the addition of a small amount (2%) of soy TVP to beef burgers was not enough to increase cooking yield (which was ~75%) or decrease shrinkage.

Shape retention (i.e., diameter and thickness changes) during cooking is desirable in terms of the mechanization of a process and the general appearance of the product [40]. Changes in shape including shrinkage are part of a normal process during cooking due to the denaturation of the meat proteins leading to moisture and fat loss and, as a consequence, changes in thickness and diameter [41]. As shown in Figure S1 and Figure 1B, the diameter change was lower for both TVP-containing hybrid burgers, with values of ~15%, compared to the real beef burger with a diameter change of 23.9% after cooking. Similar results were obtained by Gujral et al. [41] where adding 20% of soy TVP created a proportional decrease in the shrinkage of goat meat patties from 24.9% (0% TVP) to 8.9%. For thickness change, the addition of the faba bean TVP, but not the commercial TVP, decreased the value compared to the real beef burger. The difference between the two TVPs may be related to the denaturation temperature of each pulse where the faba bean has a slightly higher temperature range (95 °C to 100 °C) compared to pea (85 °C to 95 °C) [43]. Samard et al. [18] also found that TVP (soy protein, wheat gluten, and corn starch) burgers had a lower cooking loss, higher moisture retention, and lower thickness and diameter change compared to a commercial beef burger.

The texture profile results in Table 6 showed that the burger containing faba bean TVP had similar texture attributes compared to the real beef burger that did not contain TVP. In contrast, the commercial TVP-containing burger had > 40% higher hardness and gumminess values compared to both the faba bean TVP burger and the control beef burger. Similar to the results for the commercial pea TVP burger, Gujral et al. [41] reported that the addition of 20% soy TVP increased the hardness, cohesiveness, gumminess, and chewiness of baked goat burgers. Carvalho et al. [42] reported burgers containing 2% soy TVP to have a hardness value of 20.5 N and a cohesiveness value of 64%. Interestingly, in the previous texture measurements of the rehydrated TVP, the CTVP-1 had lower hardness and gumminess than the T4 faba bean TVP (although not statistically significant due to the large variation in the texture measurements). In those measurements, the TVP was rehydrated in excess water (i.e., more water than the TVP could absorb) whereas in the burger formulation, the TVP was rehydrated at a 1:1.5 ratio of TVP/water. This ratio is well below the amount of water the TVP can absorb. It is hypothesized that, in the burger, the CTVP-1, due to its high rehydration ratio (545.9% versus 308.9% for T4 faba bean TVP), absorbed much of the liquid in the meat leading to a dryer and therefore harder burger surface after cooking. Similarly, in a study on the effects of barrel temperature and MC on TVP properties, Samard et al. [18] reported that the TVP with the highest rehydration ratio and integrity index produced burgers with the highest hardness texture value. However, Kassama et al. [44] reported that there was no change in the hardness values of cooked beef burgers containing 5% soy TVP despite the TVP-containing raw burgers having a two-fold higher WHC.

### 3.11. Cooking and Texture Properties for Vegan Burgers

Images of the cooked vegan burgers are given in Figure S3. The appearance and texture of the cooked patty are highly influenced by the cooking properties which all relate to the sensory aspects of a meatless burger [18]. Compared to the hybrid formulations, the amount of TVP was doubled and used as the main ingredient in vegan burgers (see Section 2 and Table S1). A commercial vegan burger (beyond burger) was used as a control to compare to the TVP burgers in this study. The cooking properties of the three vegan burgers are presented in Figure 1C. The cooking yield of the faba bean TVP burger, at 69.5%, was lower than that of the beyond burger at 73.5%, whereas the commercial TVP burger had the highest cooking yield at 75.0%. For moisture retention, once again, the faba bean TVP burger had the lowest value at 33.5% whereas the commercial TVP burger and beyond burger were similar (39.0 and 40.6%, respectively). Corresponding to the lower cooking yield and moisture retention values, the faba bean TVP burger had the greatest diameter changes of all the burgers whereas the beyond burger had the smallest (Figure 1D). However, despite the high cooking yield and moisture retention and low diameter change, the beyond burger had over twice the thickness change as the TVP (commercial or faba bean) burgers.

The texture profile analyses of all three vegan burgers are given in Table 6. Overall, the faba bean TVP burger was more similar to the beyond burger than the commercial TVP burger when considering all six texture properties. There were large differences in the hardness values of the three burgers where commercial TVP was the hardest (51.4 N), followed by the faba bean TVP burger (31.8 N), and finally the beyond burger (24.3 N). Despite having the lowest moisture retention after cooking, the faba bean TVP burger was not the hardest. In contrast, Botella-Martinez et al. [45] reported TVP-containing burgers with lower moisture retention to be harder but also attributed this to ingredient interactions (i.e., crosslinking) and fiber and starch content. Gumminess and chewiness followed the same trend with the commercial TVP burger having over double the values as the beyond burger whereas the faba bean TVP burger was in the middle. For springiness, the faba bean TVP burger had a similar value as the beyond burger whereas the commercial TVP burger was higher. The two TVP burgers were similar in their cohesiveness and resilience values but higher than the beyond burger. Changes in extrusion variables can influence the strength and structure of the TVP protein network and, as a consequence, change the textural attributes [18]. Samard et al. [18] reported that an increase in temperature from 130 °C to 150 °C decreased the springiness and cohesiveness of burgers prepared with the TVPs whereas an increase in MC during extrusion (from 40% to 50%) in general resulted in increased springiness, cohesiveness, and hardness of TVP burgers.

## 4. Conclusions

This study produced texturized vegetable proteins (TVPs) from a faba bean protein blend (70% isolate + 30% concentrate) under different extrusion conditions (feed MCs, barrel temperature profiles, and screw speeds). Overall, the combination of extrusion variables can be used to tailor the protein structure based on the desired TVP properties. Independently, higher MCs or temperatures decreased die pressure, torque, and SME whereas increasing the screw speed increased die pressure and decreased the other two parameters. Lower feed MCs or higher extrusion temperatures produced darker-colored TVPs. The OHC increased after extrusion but did not follow clear trends based on the extrusion parameters whereas the WHC of the TVP flour was improved by a decrease in screw speed. The RR was increased by a decrease in MC. BD was affected by all extruder parameters. Higher screw speed or lower MC resulted in TVP with lower gumminess and chewiness values whereas hardness was only affected by feed MC. The TVP produced using moderate temperature (125 °C) and screw speed (300 rpm) combined with lower feed moisture content (30%) was selected for application in burgers due to its high rehydration ratio and balanced texture profile (compared to commercial pea TVPs tested). The faba bean TVP improved the cooking properties of a hybrid burger (25% reduction in beef) relative to the control beef burger as it better retained its shape, had a higher cooking yield, and retained more moisture after cooking, all while giving similar texture properties to the control. In the vegan burger, the faba bean TVP did not perform as well as the controls (commercial TVP or commercial vegan burger) in terms of cooking properties but had intermediate texture properties. Additional studies are needed to determine the sensory properties and overall acceptance of hybrid and vegan burgers. After this, optimizing processing parameters for the faba bean TVP on an industrial-scale extruder would be recommended. Overall, faba bean TVP is a high-protein plant-based ingredient that will be useful in many food applications (e.g., ground beef analogs, chicken nugget/strip analogs, and vegetarian/vegan dishes) due to its functionality.

## Figures and Tables

**Table 1:** Extrusion conditions for faba bean-based TVPs.

Sample	Barrel Temperature Profile (°C)	Moisture Content (%)	Screw Speed (rpm)
T1	75-95-115-120-125	35	200
T2	75-95-115-120-125	35	300
T3	75-95-115-120-125	35	400
T4	75-95-115-120-125	30	300
T5	75-95-115-120-125	40	300
T6	90-110-130-135-140	35	300
T7	60-80-100-105-110	35	300

**Table 2:** The effect of die temperature, screw speed, and moisture content on the response variable within the extruder for faba bean-based TVPs.

Sample	Die Pressure (kPa)	Torque (%)	SME (Wh/Kg)
T1	3234.7 ± 126.2 ^d^	56.9 ± 3.1 ^a^	118.4 ± 8.3 ^a^
T2	3558.1 ± 394.6 ^d^	28.8 ± 1.0 ^c^	89.9 ± 3.5 ^c^
T3	3962.5 ± 175.1 ^c^	25.2 ± 1.0 ^d^	105.1 ± 6.4 ^b^
T4	5337.2 ± 315.1 ^b^	32.5 ± 1.8 ^b^	109.2 ± 6.6 ^b^
T5	2163.2 ± 92.6 ^f^	25.0 ± 1.0 ^d^	72.0 ± 3.7 ^d^
T6	2628.2 ± 194.9 ^e^	23.3 ± 0.3 ^d^	72.8 ± 3.3 ^d^
T7	5761.8 ± 264.4 ^a^	33.6 ± 0.6 ^b^	104.8 ± 7.2 ^b^

Notes: Treatments (T) are as follows: T1: 125 °C, 35% moisture content (MC), 200 rpm (screw speed); T2: 125 °C, 35% MC, 300 rpm; T3: 125 °C, 35% MC, 400 rpm; T4: 125 °C, 30% MC, 300 rpm; T5: 125 °C, 40% MC, 300 rpm; T6: 140 °C, 35% MC, 300 rpm; and T7: 110 °C, 35% MC, 300 rpm. Samples with the same superscript letter within a column are not significantly different (*p* > 0.05).

**Table 3:** Protein content and physical properties of faba bean-based TVPs and commercial pea TVPs.

Sample	Protein Content (%)	Water Activity	Color		
			L *	A *	B *
Raw faba bean protein	82.8 ± 0.1 ^de^	0.3 ± 0.00 ^a^	86.5 ± 0.0 ^a^	4.2 ± 0.0 ^f^	16.9 ± 0.3 ^g^
T1	83.0 ± 0.1 ^de^	0.19 ± 0.01 ^de^	71.5 ± 0.9 ^d^	9.4 ± 0.1 ^e^	30.40 ± 0.2 ^e^
T2	84.1 ± 0.3 ^a^	0.20 ± 0.02 ^cd^	71.0 ± 0.9 ^de^	9.9 ± 0.3 ^d^	32.2 ± 0.8 ^d^
T3	82.7 ± 0.3 ^e^	0.19 ± 0.00 ^de^	70.6 ± 0.7 ^de^	10.2 ± 0.1 ^d^	32.7 ± 0.2 ^cd^
T4	83.2 ± 0.3 ^cd^	0.18 ± 0.01 ^ef^	68.6 ± 1.2 ^f^	10.7 ± 0.3 ^c^	33.4 ± 0.5 ^bc^
T5	83.3 ± 0.4 ^cd^	0.21 ± 0.00 ^bc^	71.2 ± 0.3 ^de^	9.4 ± 0.6 ^e^	31.0 ± 0.9 ^e^
T6	83.9 ± 0.2 ^ab^	0.21 ± 0.01 ^cd^	68.2 ± 0.3 ^f^	10.8 ± 0.1 ^c^	33.9 ± 0.2 ^b^
T7	83.5 ± 0.2 ^bc^	0.23 ± 0.00 ^b^	70.4 ± 0.3 ^e^	10.0 ± 0.1 ^d^	32.8 ± 0.1 ^cd^
CTVP-1	80.6 ± 0.1 ^f^	0.15 ± 0.00 ^g^	73.6 ± 0.2 ^c^	12.8 ± 0.1 ^a^	35.8 ± 0.2 ^a^
CTVP-2	75.2 ± 0.3 ^g^	0.15 ± 0.01 ^fg^	77.0 ± 0.3 ^b^	11.9 ± 0.3 ^b^	27.2 ± 0.8 ^f^

Notes: Raw faba bean protein, 70% of faba bean protein isolate + 30% of faba bean protein concentrate. Treatments (T) are as follows: T1: 125 °C, 35% moisture content (MC), 200 rpm (screw speed); T2: 125 °C, 35% MC, 300 rpm; T3: 125 °C, 35% MC, 400 rpm; T4: 125 °C, 30% MC, 300 rpm; T5: 125 °C, 40% MC, 300 rpm; T6: 140 °C, 35% MC, 300 rpm; and T7: 110 °C, 35% MC, 300 rpm. CTVP, commercial pea texturized vegetable protein. Samples with the same superscript letter within a column are not significantly different (*p* > 0.05).

**Table 4:** Functional properties of faba bean-based TVPs and commercial pea TVPs.

Sample	* Water Holding Capacity (g Water/g Protein)	* Oil Holding Capacity (g Oil/g Protein)	Rehydration Ratio (%)	Bulk Density (g/mL)
Raw faba bean protein	3.17 ± 0.03 ^cdef^	0.99 ± 0.03 ^g^	NA	NA
T1	3.78 ± 0.11 ^ab^	1.69 ± 0.06 ^bc^	296.9 ± 11.2 ^cd^	0.36 ± 0.01 ^c^
T2	3.54 ± 0.11 ^bc^	1.61 ± 0.07 ^bc^	279.7 ± 13.7 ^d^	0.38 ± 0.01 ^b^
T3	3.30 ± 0.06 ^cde^	1.59 ± 0.04 ^cd^	290.3 ± 6.8 ^d^	0.39 ± 0.02 ^b^
T4	3.03 ± 0.10 ^ef^	1.45 ± 0.07 ^e^	308.9 ± 9.4 ^c^	0.41 ± 0.00 ^a^
T5	2.95 ± 0.11 ^f^	1.32 ± 0.03 ^f^	155.1 ± 15.4 ^f^	0.30 ± 0.01 ^e^
T6	3.38 ± 0.15 ^cd^	1.50 ± 0.04 ^de^	237.5 ± 3.9 ^e^	0.42 ± 0.01 ^a^
T7	3.15 ± 0.15 ^def^	1.60 ± 0.05 ^bcd^	235.2 ± 8.0 ^e^	0.34 ± 0.00 ^d^
CTVP-1	3.88 ± 0.05 ^ab^	1.68 ± 0.03 ^bc^	545.9 ± 19.6 ^a^	0.20 ± 0.00 ^f^
CTVP-2	4.10 ± 0.05 ^a^	2.83 ± 0.12 ^a^	390.1 ± 14.1 ^b^	0.11 ± 0.00 ^g^

* Measured on the milled TVP. Notes: Raw faba bean protein, 70% of faba bean protein isolate + 30% of faba bean protein concentrate. Treatments (T) are as follows: T1: 125 °C, 35% moisture content (MC), 200 rpm (screw speed); T2: 125 °C, 35% MC, 300 rpm; T3: 125 °C, 35% MC, 400 rpm; T4: 125 °C, 30% MC, 300 rpm; T5: 125 °C, 40% MC, 300 rpm; T6: 140 °C, 35% MC, 300 rpm; and T7: 110 °C, 35% MC, 300 rpm. CTVP, commercial pea texturized vegetable protein; NA, not applicable. Samples with the same superscript letter within a column are not significantly different (*p* > 0.05).

**Table 5:** Texture profile of faba bean-based TVPs and commercial pea TVPs.

Sample	Hardness (N)	Springiness (%)	Cohesiveness (%)	Gumminess (N)	Chewiness (N)	Resilience (%)
T1	5.7 ± 0.6 ^bcd^	76.8 ± 3.4 ^bc^	63.0 ± 2.3 ^b^	3.6 ± 0.4 ^bc^	2.8 ± 0.4 ^ab^	33.9 ± 2.1 ^bc^
T2	3.7 ± 0.9 ^e^	70.1 ± 2.8 ^de^	56.8 ± 2.7 ^de^	2.3 ± 0.9 ^d^	1.7 ± 0.7 ^c^	29.2 ± 2.5 ^e^
T3	4.3 ± 0.5 ^cde^	70.4 ± 1.5 ^cde^	58.5 ± 1.0 ^d^	2.5 ± 0.3 ^cd^	1.8 ± 0.3 ^c^	30.8 ± 1.1 ^de^
T4	4.5 ± 0.2 ^cde^	73.1 ± 1.5 ^cd^	62.5 ± 2.1 ^bc^	2.8 ± 0.1 ^cd^	2.1 ± 0.1 ^bc^	34.4 ± 1.3 ^b^
T5	9.9 ± 0.6 ^a^	65.1 ± 2.1 ^e^	53.9 ± 2.3 ^e^	5.4 ± 0.2 ^a^	3.6 ± 0.2 ^a^	33.6 ± 1.5 ^bcd^
T6	7.2 ± 0.6 ^b^	68.4 ± 1.6 ^de^	58.7 ± 1.0 ^cd^	4.2 ± 0.5 ^ab^	2.9 ± 0.4 ^ab^	33.0 ± 0.5 ^bcd^
T7	5.9 ± 0.4 ^bc^	67.9 ± 4.8 ^de^	56.5 ± 1.2 ^de^	3.3 ± 0.3 ^bcd^	2.7 ± 0.2 ^bc^	31.6 ± 1.1 ^cde^
CTVP-1	2.6 ± 0.0 ^e^	88.7 ± 1.7 ^a^	75.9 ± 1.0 ^a^	2.0 ± 0.0 ^cd^	1.8 ± 0.1 ^bc^	40.7 ± 1.0 ^a^
CTVP-2	3.6 ± 0.1 ^de^	82.2 ± 1.8 ^ab^	66.3 ± 3.0 ^b^	2.4 ± 0.2 ^cd^	1.9 ± 0.2 ^c^	36.8 ± 1.5 ^ab^

Notes: Treatments (T) are as follows: T1: 125 °C, 35% moisture content (MC), 200 rpm (screw speed); T2: 125 °C, 35% MC, 300 rpm; T3: 125 °C, 35% MC, 400 rpm; T4: 125 °C, 30% MC, 300 rpm; T5: 125 °C, 40% MC, 300 rpm; T6: 140 °C, 35% MC, 300 rpm; and T7: 110 °C, 35% MC, 300 rpm. CTVP, commercial pea texturized vegetable protein. Samples with the same superscript letter within a column are not significantly different (*p* > 0.05).

**Table 6:** Texture profile of burger patties.

	Hardness (N)	Springiness (%)	Cohesive-ness (%)	Gumminess (N)	Chewiness (N)	Resilience (%)
Hybrid						
CTVP-1	27.1 ± 0.9 ^a^	118.2 ± 23.4 ^a^	90.9 ± 1.9 ^a^	24.6 ± 1.3 ^a^	29.6 ± 7.3 ^a^	66.5 ± 0.8 ^a^
TVP-T4	18.8 ± 0.7 ^b^	119.5 ± 7.5 ^a^	92.6 ± 0.6 ^a^	17.4 ± 0.6 ^b^	20.7 ± 2.1 ^a^	67.2 ± 0.5 ^a^
Real beef	19.2 ± 4.8 ^b^	103.3 ± 15.0 ^a^	87.5 ± 6.5 ^a^	17.5 ± 4.7 ^b^	21.2 ± 2.5 ^a^	63.2 ± 3.9 ^a^
Vegan						
CTVP-1	51.4 ± 0.0 ^a^	107.2 ± 5.4 ^a^	88.4 ± 0.2 ^a^	45.4 ± 0.1 ^a^	48.6 ± 2.6 ^a^	58.1 ± 0.3 ^a^
TVP-T4	31.8 ± 0.8 ^b^	94.1 ± 1.2 ^b^	88.5 ± 0.2 ^a^	28.1 ± 0.7 ^b^	26.5 ± 1.0 ^b^	54.4 ± 1.1 ^a^
Beyond burger	24.3 ± 0.5 ^c^	83.3 ± 5.9 ^b^	76.7 ± 3.3 ^b^	18.4 ± 0.4 ^c^	15.3 ± 1.3 ^c^	32.0 ± 2.7 ^b^

Notes: For each texture property, samples with the same superscript letter within hybrid or vegan formulations are not significantly different (*p* > 0.05). CTVP-1 = commercial pea TVP (CTVP-1); TVP-T4 = faba bean TVP, 125 °C, 30% moisture content, 300 rpm (screw speed).

## Data Availability

The original contributions presented in this study are included in the article/Supplementary Materials. Further inquiries can be directed to the corresponding author.

## Supplementary Materials

The following supporting information can be downloaded at https://www.mdpi.com/article/10.3390/foods14040547/s1, Table S1: Burger patty formulations; Table S2: Cooking properties of hybrid and vegan burgers containing commercial TVP (CTVP-1) or faba bean TVP (TVP-T4: 125 °C, 30% MC, 300 rpm); Figure S1: Scanning electron microscopy images of raw faba bean isolate and concentrate with different magnification; Figure S2: Images of cooked real beef burger and hybrid burgers containing TVP; Figure S3: Images of cooked vegan burgers.
