# Supplementary material for: Effect of Extrusion Conditions on the Characteristics of Texturized Vegetable Protein from a Faba Bean Protein Mix and Its Application in Vegan and Hybrid Burgers
Source: Foods. 2025 Feb 7;14(4):547. doi: 10.3390/foods14040547 (PMC11854062; doi:10.3390/foods14040547)
Supplement: Supplementary file 1 [file foods-14-00547-s001.zip › foods-3443599-supplementary.pdf]

Supplementary

Table S1. Burger patty formulations

Ingredient	Control (%)	Hybrid (%)	Vegan (%)
Lean ground beef (16% fat)	91	68.15	0
TVP (unhydrated)	0	10	20
Water	7.7	15.3	60
Black pepper	0.3	0.3	0.3
Salt	1	1	1
Vegan food colour & flavour	0	0.85	3.2
Canola oil	0	4.4	13
Methylcellulose	0	0	2.5
Total	100	100	100

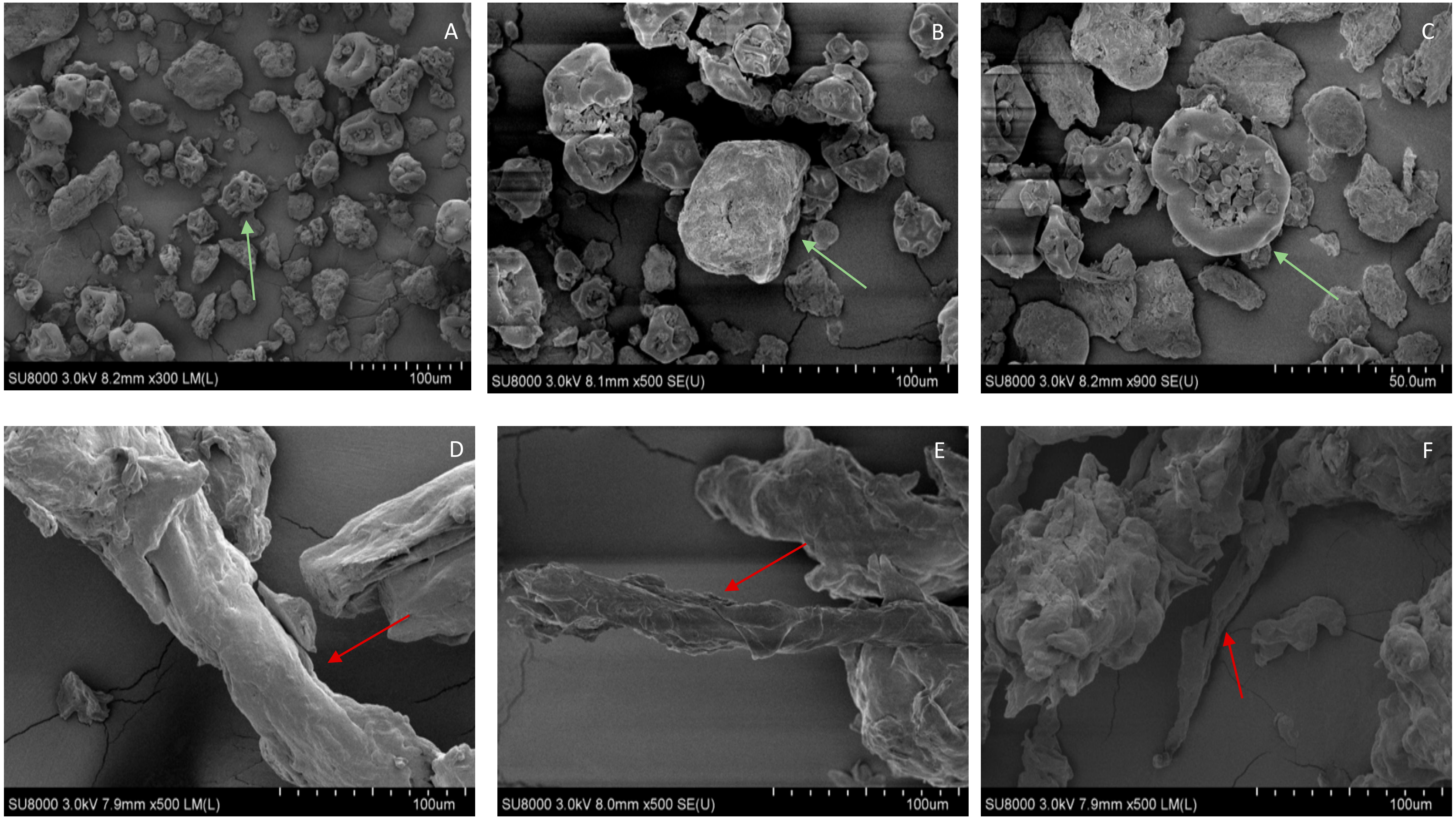
A
SU8000 3.0kV 8.2mm x300 LM(L)
100um
B
SU8000 3.0kV 8.1mm x500 SE(U)
100um
C
SU8000 3.0kV 8.2mm x900 SE(U)
50.0um
D
SU8000 3.0kV 7.9mm x500 LM(L)
100um
E
SU8000 3.0kV 8.0mm x500 SE(U)
100um
F
SU8000 3.0kV 7.9mm x500 LM(L)
100um

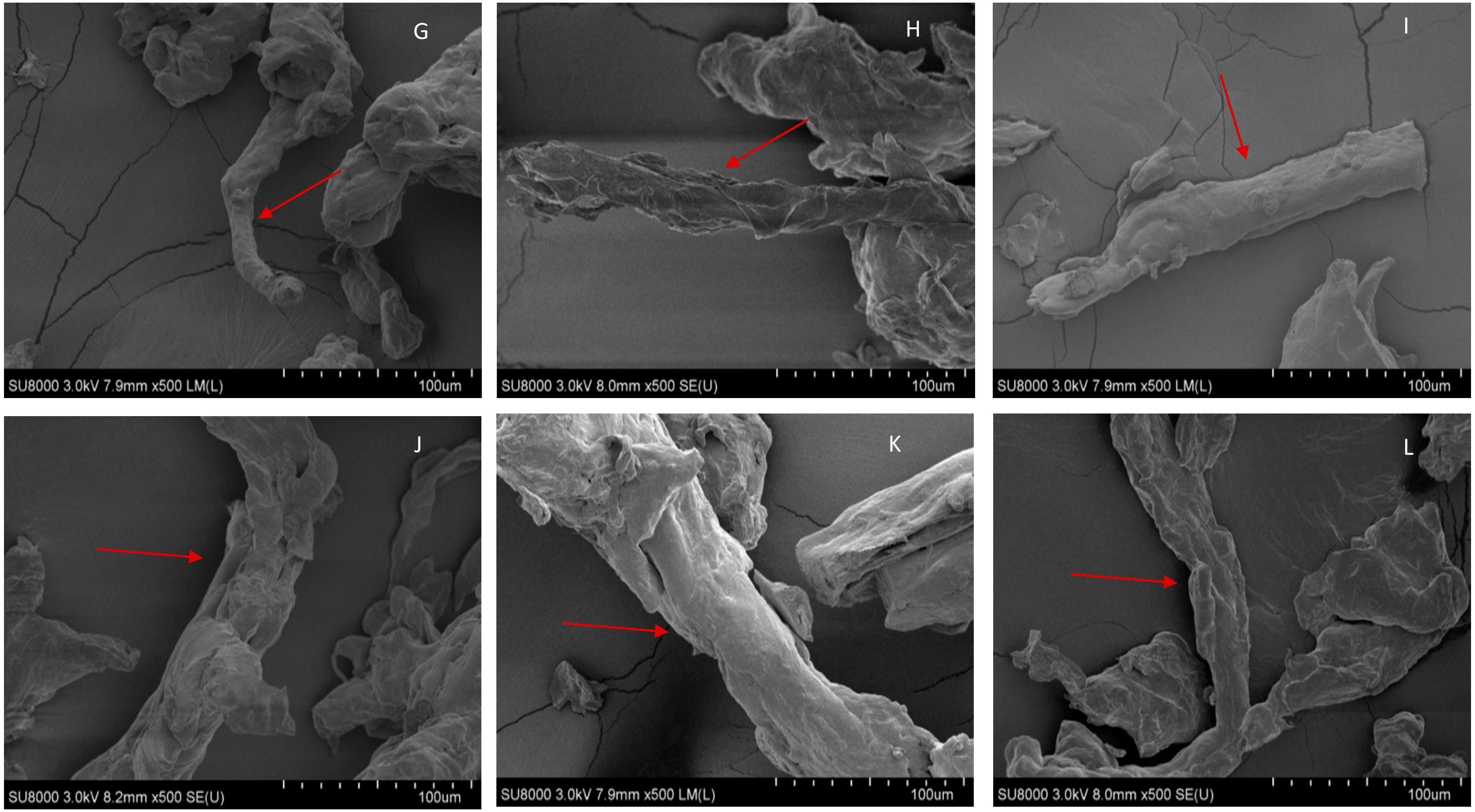

Figure S1. Scanning electron microscopy images of raw faba bean isolate and concentrate with different magnification, (A) 300×, (B) 500× and (C) 900×; and (D-L) intact faba bean-based TVP (500× magnification): as function of feed moisture content, (D) 30% (E) 35% and (F) 40% at constant temperature of 125ºC; as a function of die temperature (G) 110ºC (H) 125ºC and (I) 140ºC at constant feed moisture of 35%; and as a function of screw speed (J) 200 rpm (K) 300 rpm and (L) 400 rpm at constant temperature of 125ºC. A-C: arrows indicate the globular structure and D-L: arrows indicate the elongated structure. Note: the following images are intentional duplicates in order to follow the specific extrusion process parameters (i.e. function of moisture content, temperature, or screw speed): D and K; E and H.

Table S2. Cooking properties of hybrid and vegan burgers containing commercial TVP (CTVP-1) or faba bean TVP (TVP-T4: 125ºC, 30% MC, 300 rpm).

	**Cooking yield (%)**	**Moisture retention (%)**	**Diameter change (%)**	**Thickness change (%)**
Hybrid				
CTVP-1	83.0 ± 1.0^{a}	49.3 ± 0.2^{a}	15.1 ± 1.9^{b}	24.5 ± 0.4^{a}
TVP-T4	81.0 ± 2.0^{a}	47.5 ± 1.5^{a}	14.7 ± 1.0^{b}	13.7 ± 0.3^{b}
Real beef	68.7 ± 1.2^{b}	42.7 ± 3.3^{b}	23.9 ± 5.5^{a}	22.8 ± 0.7^{a}
Vegan				
CTVP-1	75.0 ± 0.2^{a}	39.0 ± 0.2^{a}	9.0 ± 0.5^{b}	5.4 ± 1.0^{b}
TVP-T4	69.5 ± 0.7^{c}	33.5 ± 1.1^{b}	11.3 ± 0.2^{a}	7.0 ± 0.3^{b}
Beyond burger	73.5 ± 0.5^{b}	40.5 ± 0.3^{a}	7.1 ± 1.2^{c}	16.5 ± 3.5^{a}

Notes: For each cooking property, samples with the same superscript letter within the hybrid or vegan formulation are not significantly different ($p>0.05$).

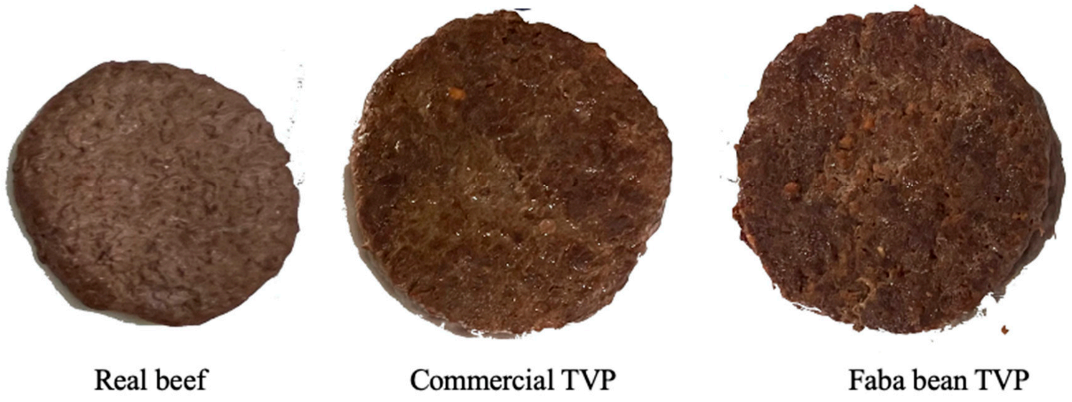

Figure S2. Images of cooked real beef burger and hybrid burgers containing TVP.

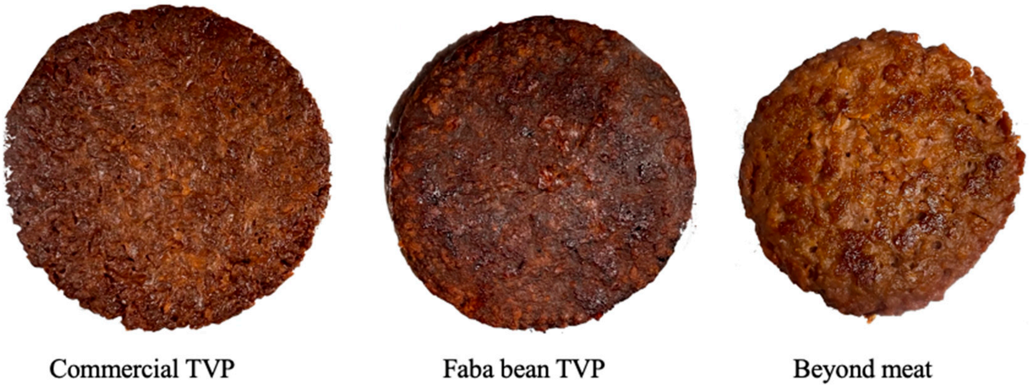

Figure S3. Images of cooked vegan burgers.
